# Evaluation of Antioxidant Effects of Pumpkin (*Cucurbita pepo* L.) Seed Extract on Aging- and Menopause-Related Diseases Using Saos-2 Cells and Ovariectomized Rats

**DOI:** 10.3390/antiox13020241

**Published:** 2024-02-16

**Authors:** Joohee Oh, Sookyeong Hong, Seong-Hee Ko, Hyun-Sook Kim

**Affiliations:** Department of Food and Nutrition, College of Human Ecology, Sookmyung Women’s University, Seoul 04310, Republic of Korea

**Keywords:** antiaging, menopause, pumpkin seed, oxidative stress, antioxidants, cardiovascular diseases, osteoporosis

## Abstract

Aging and menopause are associated with oxidative stress and inflammation. Here, we evaluated the antioxidant properties of pumpkin (*Cucurbita pepo* L.) seed extract and assessed its ameliorative effects on aging- and menopause-related diseases using Saos-2 cells and ovariectomized rats. The seed extract had bioactive components that exhibited antioxidant activity. The extract increased the alkaline phosphatase (ALP) activity of Saos-2 cells. The oral administration of the extract to ovariectomized rats for 12 weeks decreased their body weight, fat weight, and cardiac risk indices. It also contributed to reductions in the levels of reactive oxygen species, oxidative stress, and inflammation, as assessed by measuring the serum levels of malondialdehyde and analyzing gene expression in rats. Furthermore, the administration of the extract also promoted an enhancement of the transcription of nuclear factor erythroid 2-related factor (*Nrf2*), heme oxygenase-1 (*Ho-1*), and catalase (*Cat*), involved in antioxidant activity; endothelial nitric oxide synthase (*eNos*), involved in vasculoprotective activity; and PR/SET domain 16 (*Prdm16*) and peroxisome proliferator-activated receptor-gamma coactivator *(Pgc1α*), involved in brown adipogenesis and thermogenesis. Our results using ovariectomized rats show that pumpkin seed extract may have ameliorative effects on menopause-related diseases by increasing ALP activity, evaluating the antioxidant system, ameliorating oxidative stress and thermogenesis, and enhancing lipid profiles.

## 1. Introduction

Aging is a natural physiological and pathological metabolic phenomenon that causes the degeneration of the body and the deterioration of physical functions that are crucial for the survival and maintenance of life [1,2]. Menopause, which is defined as the end of menstrual cycles, is a natural reproductive aging process and is closely associated with aging. Menopause is also linked with an increased risk of endocrine disorders such as dyslipidemia, cardiovascular diseases, hypertension, osteoporosis, vascular inflammation, and sleep disorders, which are associated with a reduction in estrogen levels [3,4,5]. Moreover, in addition to endocrine disorders, body weight and lipid metabolism are markedly altered in post-menopausal women [6]. Clinical epidemiological studies have revealed that postmenopausal women and ovariectomized patients are at an increased risk of cardiovascular diseases caused by estrogens deficiency-induced oxidative stress [7].

Under oxidative stress, aerobic respiration contributes to the excessive production of free radicals, resulting in the accumulation of oxidative damage and leading to aging and ultimately death [8]. The imbalance in the production and removal of reactive oxygen species (ROS) induces oxidative stress [9,10]. Oxidative stress causes lipid peroxidation, cDNA damage, and protein denaturation, which may lead to the dysfunction of physiological processes in the body [11].

**Figure 1:**
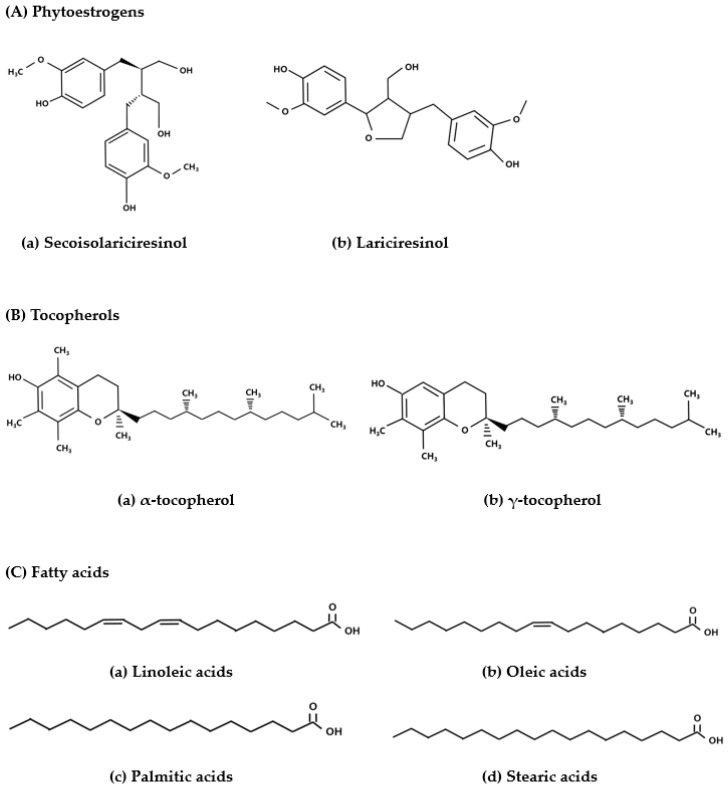
Structure of the phytoestrogens, antioxidants tocopherols, and fatty acids present in pumpkin seeds [12].

The pumpkin plant (*Curcubita* sp.) is distributed throughout Asia, with *Cucurbita pepo* L. being the most common species [12]. Pumpkin seeds are functional food rich in bioactive compounds that exhibit antidiabetic, antioxidant, antitumor, and antidepression properties [13]. Phytoestrogens form a large group of heterocyclic phenols that have functions and structures similar to those of 17β-estradiol and synthetic estrogens [14]. The seeds also contain lignan phytoestrogens (256 mg/100 g), such as secoisolariciresinol and lariciresinol, and several types of antioxidants, including tocopherols (16 mg/100 g), and are a rich source of fatty acids, such as linoleic, oleic, palmitic, and stearic acids (Figure 1) [12,15]. These phytoestrogens and tocopherols exhibit antioxidant properties, whereas the essential fatty acids may potentially have beneficial effects with respect to menopausal syndromes caused by estrogens deficiency and in protecting against cardiovascular diseases [16,17,18].

Hormone replacement therapy (HRT) is used to treat harmful conditions associated with estrogens deficiency; however, it causes adverse effects such as internal bleeding and increases the risk of breast cancer and other complications [19]. Phytoestrogens can provide a safer alternative therapy by replacing estrogens function [20]. Pumpkin seeds, which contain phytoestrogens and antioxidants, have a potential use in such alternative therapy.

Considering the presence of antioxidants and phytoestrogens in pumpkin seeds, we hypothesized that an extract of pumpkin seeds could potentially contribute to regulating lipid metabolism and oxidative stress, thereby mitigating cardiovascular risk, thermogenesis, and osteoporosis. To test this hypothesis, we investigated the ameliorating effect of pumpkin seed extract on aging- and menopause-related diseases in ovariectomized rats, which are used as a valuable model for estrogen deficiency-induced diseases.

## 2. Materials and Methods

### 2.1. Materials

Pumpkin seeds (*C. pepo* L.) were purchased from an agricultural product market in Gangjin-gun, Jeollanam-do, Korea. The seed coat was peeled off, and the seeds were ground to a powder using a grinder.

### 2.2. Preparation of Pumpkin Seed Extract

Pumpkin seed powder (100 g) was dissolved in 1 L of distilled water (DW) and incubated overnight at 20–22 °C. The mixture was centrifuged at 1902× *g* for 30 min and filtered through filter paper (5–8 µm, Qualitative, no. 2, Hyundai Micro, Seoul, Republic of Korea). The filtrate was concentrated in a rotary vacuum evaporator (N-21NS; EYELA, Tokyo, Japan) at 40 °C, freeze-dried at −90 °C (Operon FDUT-8806, Gimpo, Gyeonggi, Republic of Korea), and stored at −20 °C until further use.

### 2.3. Antioxidant Content Analysis

#### 2.3.1. Quantitation of Total Polyphenol Content

Total polyphenol content in the pumpkin seed extract was determined using the Folin–Ciocalteu method [21]. The samples were dissolved in DW at a concentration of 100 mg/mL. The extracts were added to different test tubes and mixed with 1 mL 2% Na_2_CO_3_ by vortexing. After 5 min, 200 µL of Folin–Ciocalteu reagent was added to the tubes, and the mixture was incubated at room temperature for 30 min in the dark. The absorbance of the solution was measured at 750 nm using an Epoch Microplate Spectrophotometer (Biotek Inc., Winooski, VT, USA). A standard curve was prepared to determine the total polyphenol content, which was expressed as gallic acid equivalents (mg GAE/g) [22].

#### 2.3.2. Quantitation of Total Flavonoid Content

The total flavonoid content of the pumpkin seed extract was measured using the Davis method [23]. The samples were dissolved in DW at a concentration of 100 mg/mL. Further, 100 µL of the extract was mixed with 5 mL of 90% diethylene glycol by vortexing, and then, 100 µL of 4 N NaOH was added. The mixture was incubated in a water bath at 30 °C. The absorbance of the solution was measured at 420 nm. The total flavonoid content was determined using a standard curve prepared using quercetin and expressed as milligrams of quercetin equivalents (QE) per gram [24].

### 2.4. Analysis of the Radical-Scavenging Capacity

Free radical-scavenging activity was measured using the 2,2-diphenyl-1-picrylhydrazyl (DPPH) method as described previously [25]. DPPH solution was prepared by dissolving DPPH (0.1 mM) in methanol, and different concentrations of DPPH were added to the samples. The absorbance of the DPPH solution was measured at 517 nm, and when it reached 0.95–0.99, 200 µL of pumpkin seed extract and 800 µL of DPPH solution were mixed, vortexed, and kept in the dark at room temperature for 20 min. Absorbance was measured at 517 nm. Values were calculated using an ascorbic acid calibration curve. The DPPH radical-scavenging activity was calculated using the following equation:

DPPH inhibition (%) = [(A_blank_ − A_sample_)/A_blank_ ] × 100 (1)

2,2′-Azino-bis (3-ethylbenzothiazoline-6-sulfonic acid) (ABTS) radical-scavenging activity was measured as described previously [26]. The ABTS solution (radical stock solution, RSS) was prepared by mixing 7 mM ABTS and 2.45 mM potassium persulfate in DW at a 1:1 ratio. The mixture was then stored in the dark at room temperature for 12–16 h. Thereafter, 4.3 mL of RSS was diluted with 100 mL of phosphate-buffered saline (PBS) to obtain an absorbance of 0.700 ± 0.02 at 734 nm. Then, 50 µL of sample and 950 µL of diluted reagent were mixed and incubated in the dark for 30 min. The absorbance of the mixture was measured at 734 nm. Values were calculated using an ascorbic acid calibration curve. Percentage of inhibition was calculated using the following equation:

ABTS inhibition (%) = [(A_blank_ ± A_sample_)/A_blank_ ] × 100 (2)

### 2.5. Gas Chromatograph–Mass Spectrometer (GC-MS) Analysis

The analysis of the constituents of the pumpkin seed extract was performed using GC-MS. A 0.2 g sample of pumpkin seed extract was mixed with 2 mL of a methylation mixture (MeOH:benzene:2,2-dimethoxy-propane:H_2_SO_4_ = 39:20:5:2) and 1 mL of heptane in a 4 mL vial fitted with a Teflon cap, extracted at 80 °C for 2 h, and then filtered for experiment use. Analyses were performed using an Agilent 7890A gas chromatograph equipped with a 5977B mass selective detector (GC/MS) (Agilent Technologies, Santa Clara, CA, USA), with chromatographic separation on a DB-23 column (120 mm × 0.25 mm × 0.25 μm; Agilent). The temperature of injector was set at 250 °C, into which a 1 μL volume of sample was injected in the split mode with a ratio of 10:1. The carrier gas was helium, circulated at a constant flow rate of 2 mL/min. The GC oven temperature was initially set to 80 °C for 1.5 min, and thereafter increased to 110 °C for 2 min at 30 °C/min and then to 200 °C for 8 min at 15 °C/min, to 215 °C for 8 min at 1 °C/min, and finally to 250 °C, at which it was held for 3 min [27]. Scan mode was used in the range of 29–550 *m*/*z* for 62 min. Single compounds were identified by comparing mass spectra with NIST17 mass spectral libraries (National Institute of Standards, 2017 version).

### 2.6. Cell Culture

Human Saos-2 cells were purchased from the Korean Cell Line Bank (KCLB, Seoul, Republic of Korea). The cells were passaged to fresh plates containing phenol red-free RPMI 1640 medium supplemented with 1% penicillin–streptomycin solution and 5% charcoal/dextran-stripped serum at 37 °C in an atmosphere of 5% CO_2_ to reduce the levels of steroids and promote basal proliferation. Thereafter, the medium was replaced at 2- to 3-day intervals with fresh medium containing 5% charcoal/dextran-stripped serum [28].

### 2.7. Cell Proliferation Assay

Cell proliferation was determined using 3-(4,5-dimethylthiazol-2-yl)-2,5-diphenyltetrazolium bromide (MTT; M2128, Sigma-Aldrich, St. Louis, MO, USA) assay. Saos-2 cells were seeded in 96-well plates at a density of 1 × 10^4^ cells/cm^2^ for 24 h. After the cells had reached confluence, the culture medium was replaced with medium containing pumpkin seed extract (0, 25, 62.5, or 100 µg/mL). After 48 h of incubation, the MTT solution (5 mg/mL) was added to the wells, and the plate was incubated for 4 h in a CO_2_ incubator [29]. Thereafter, 200 µL of dimethyl sulfoxide was added to each well, and the absorbance was determined at 560 nm.

### 2.8. Alkaline Phosphatase (ALP) Activity

Cells (2 × 10^5^ cells/cm^2^) were seeded in six-well plates containing phenol red-free RPMI 1640 medium supplemented with 5% charcoal/dextran-stripped serum and 1% penicillin–streptomycin solution, and incubated for 48 h. Thereafter, the culture medium was replaced with medium containing pumpkin seed extract (0, 25, 62.5, or 100 µg/mL), and the plate was incubated for 48 h [29,30]. The cells were then washed with cold PBS and homogenized with ALP assay buffer. The lysate was centrifuged at 15,814× *g* for 15 min, and the obtained supernatant was used to measure ALP activity. Activity was measured using an alkaline phosphatase assay kit (ab83369; Abcam, Cambridge, UK), following the manufacturer’s instructions.

### 2.9. Animals and Housing Conditions

Twenty-eight 9-week-old female Sprague–Dawley rats (Saeron bio, Uiwang, Gyeonggi, Republic of Korea) were housed in a controlled environment with 22 ± 1 °C temperature, 50–60% relative humidity, and a 12 h light/dark cycle. The rats were provided ad libitum access to water and a chow diet. The experimental design was approved by the Institutional Animal Care and Use Committee (IACUC) of Sookmyung Women’s University for the Care and Use of Laboratory Animals (SMWU-IACUC-2303-003-01).

### 2.10. Experimental Design of Animal Groups

After one week of adaptation, the rats were randomly divided into the following four groups (*n* = 7 for each group): Sham, Sham-operated control group; OVX, ovariectomized negative control group; OL, OVX + low-dose pumpkin seed extract; and OH, OVX + high-dose pumpkin seed extract. After fasting for one day, sham surgery (*n* = 7) and bilateral ovariectomy (*n* = 21) were performed. During surgery, a 1:1 mixture of Zoletil50 (Virbac, Carros, France) and Rompun (Bayer Healthcare Korea, Seoul, Republic of Korea) was intraperitoneally injected at doses of 100–130 µL. After two weeks of recovery, rats from all groups were administered the respective treatments through oral gavage: Sham and OVX group were administered distilled water, OL group were administered 250 mg/kg body weight (b.w.) of pumpkin seed extract, and OH group were administered 500 mg/kg b.w. of pumpkin seed extract over six days (Monday to Saturday). The oral gavage dose of pumpkin seed extract used in this study was based on that described in a previous study and which was established to have beneficial estrogenic effects [15].

### 2.11. Measurement of b.w. and Food Intake

The b.w. of each animal was recorded once per week. The amount of food intake was recorded every 2 days. The final b.w. was recorded the day before sacrifice. Food efficiency ratio (FER) was calculated using the following equation:

FER (%) = total b.w. gain (g/total food intake (g)) × 100(3)

### 2.12. Sample Preparation

After overnight fasting, the rats were euthanized with CO_2_. Blood samples were collected in 5 mL serum separator tubes and centrifuged at 1902× *g* for 30 min (Combi-514R, Hanil Co. Ltd., Seoul, Republic of Korea), and the supernatant was stored in a deep freezer (DF8517, Ilshin Laboratory Co., Ltd., Seoul, Republic of Korea) at −70 °C until use. Liver, lungs, kidneys, abdominal fat (ABF), uterus, uterine fat (UF), brown adipose tissue (BAT), and subcutaneous fat (SCF) were removed and weighed.

### 2.13. Lipid Profile

Serum triglyceride (TG) and total cholesterol (TC) levels were measured using TG-S (3I1570; Asanpharm, Hwaseong, Republic of Korea) and T-CHO (3I2020; Asanpharm, Seoul, Republic of Korea) kits, respectively. High-density lipoprotein cholesterol (HDL-C) was measured using an HDL-CHO kit (3I2030; Asanpharm, Seoul, Republic of Korea). The levels of low-density lipoprotein cholesterol (LDL-C) were determined using the Friedewald equation [31]:

LDL-C level (mg/dL) = TC level − (HDL-C level) + TG level/5(4)

### 2.14. Atherogenic Index (AI) and Cardiac Risk Indices (CRI)-I and -II

AI, CRI-I, and CRI-II were calculated using the Haglund method [32] with the following equations:

AI = (TC level − HDL-C level)/HDL-C level(5)

CRI-I = TC level/HDL-C level,(6)

CRI-II = LDL-C level/HDL-C level(7)

### 2.15. Hepatic Function

Hepatic function was assessed by determining the activity of aspartate aminotransferase (AST) and alanine aminotransferase (ALT). AST (AM103-K, Asanpharm, Seoul, Republic of Korea) and ALT (AM102, Asanpharm, Seoul, Republic of Korea) assays were performed using the respective kits, as described by the manufacturer.

### 2.16. Measurement of an Oxidative Stress Marker in the Serum

The levels of malondialdehyde (MDA), a marker of oxidative stress, in the serum were measured using an MDA ELISA kit (E-EL-0060; Elabscience, Wuhan, China), according to the manufacturer’s instructions.

### 2.17. Histological Analysis

BAT was fixed in 10% neutral buffered formalin solution (Sigma-Aldrich Co., HT501128, St. Louis, MO, USA) and embedded in paraffin. The fixed tissues were sectioned to 5 µm thickness and stained with hematoxylin and eosin (H&E) for pathological examination to assess the lipid content of BAT. The content of lipid in BAT was measured using the ImageJ software (Ver. 1.45s, National Institutes of Health, Bethesda, MD, USA).

### 2.18. Femur Function Analysis

The right femur of the rats was fixed in a 10% neutral buffered formalin solution. Bone mineral density was determined using a dual-energy X-ray bone densitometer (PIXIMUS, Lunar Corp., Madison, WI, USA). To determine the pore size of the femur section, each femur was sectioned to 5 µm thickness, and stained with H&E for pathological examination. The size of the pores in the femur was measured using the ImageJ software (Ver. 1.45s, National Institutes of Health, Bethesda, MD, USA).

### 2.19. mRNA Expression Analysis

mRNA expression in the liver, BAT, and SCF was determined using real-time quantitative-polymerase chain reaction (qRT-PCR). RNA was isolated from the liver, BAT, and SCF. Each sample was homogenized using an easy-spin™ total RNA extraction kit (17221, iNtRON Biotechnology, Inc., Seongnam, Gyeonggi, Republic of Korea). The PrimeScript™ RT reagent Kit (RR037AM; Takara Bio Inc., Shiga, Japan) was used for cDNA synthesis. The PCR products were mixed with TB Green^®^ Premix Ex Taq™ II (Tli RNaseH Plus) (RR820A; Takara Bio Inc.) and analyzed using a LightCycler 96 (Roche Molecular Systems, Inc., Pleasanton, CA, USA). For relative quantitative analysis, each data point was normalized against *Gapdh*, and the relative expression levels were determined. The primers used for qRT-PCR are listed in Table S1.

### 2.20. Statistical Analysis

Statistical analysis was performed by Prism 10.1.1 (GraphPad Software Inc., La Jolla, CA, USA). All data are shown as mean ± SD. The results for each group were analyzed using one-way analysis of variance, followed by Tukey’s multiple comparison test to determine the differences between the groups. Significance was defined as **** *p* value < 0.0001, *** *p* < 0.001, ** *p* < 0.01, or * *p* < 0.05.

## 3. Results

### 3.1. Total Polyphenol and Flavonoid Content of the Pumpkin Seed Extract

Values obtained for the total polyphenol and flavonoid contents of the pumpkin seed extracts are shown in Table 1. The total polyphenol and flavonoid contents of pumpkin seed were 784.61 ± 7.94 mg GAE/g and 0.70 ± 0.18 mg QE/g, respectively.

### 3.2. Effects of Pumpkin Seed Extract on DPPH and ABTS Radical-Scavenging Activities

**Figure 2:**
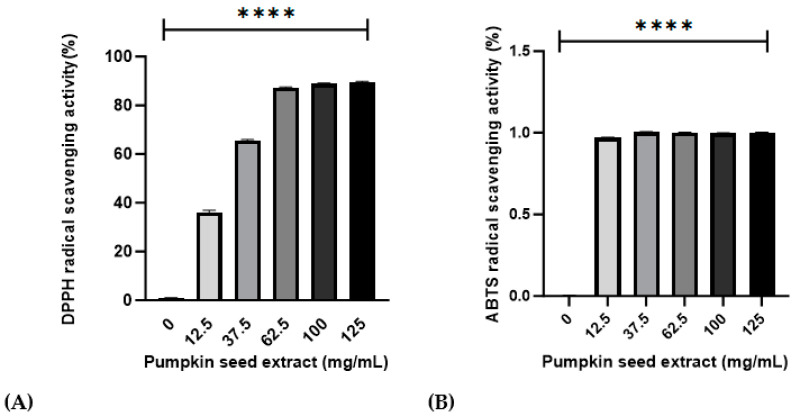
Effects of pumpkin seed extract (12.5, 37.5, 63.5, 100, and 125 mg/mL) on DPPH and ABTS radical-scavenging activities. (**A**) DPPH radical-scavenging activity (%). (**B**) ABTS radical-scavenging activity (%). Data are expressed as the mean ± SD (**** *p* < 0.0001 vs. 0 mg/mL).

Values obtained for the antioxidant activities of the pumpkin seed extract are presented in Figure 2. Relative to that of ascorbic acid, the DPPH and ABTS radical-scavenging activities were 95.31% ± 0.42% and 92.67% ± 0.20% in 100 mg/mL of pumpkin seed extract, respectively.

### 3.3. Characterization of Chemicals in Pumpkin Seed Extract by Gas Chromatograph–Mass Spectorometer (GC-MS)

**Figure 3:**
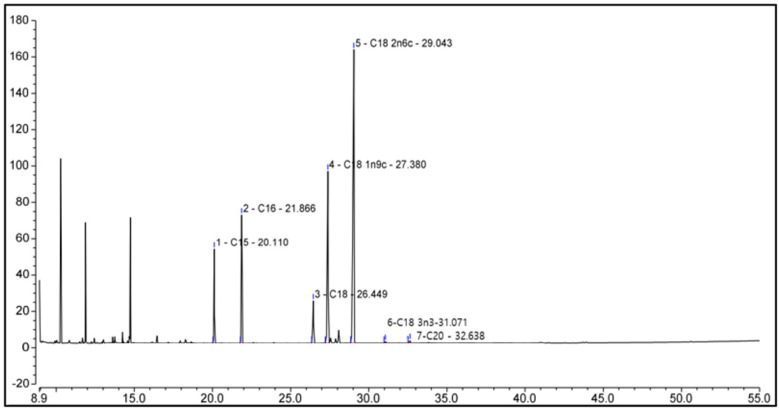
Chemicals in pumpkin seed extract. The identities of the compounds are shown in Table 2.

We performed GC-MS analysis to identify compounds present in pumpkin seed extracts, and the data of which are shown in Figure 3 and Table 2. Chromatographic profiles revealed seven compounds in the extract, among which we identified saturated, monounsaturated, and polyunsaturated fatty acids, including palmitic (hexadecanoic acid), stearic (octadecanoic acid), oleic (Omega-9), linoleic (Omega-6), alpha-linolenic (Omega-3), and arachidic (icosanoic) acids, and among which, oleic acid (23.40%), linoleic acid (39.94%), and alpha-linolenic acid (0.23%) are essential fatty acids.

### 3.4. Effects of Pumpkin Seed Extract on Cell Proliferation

**Figure 4:**
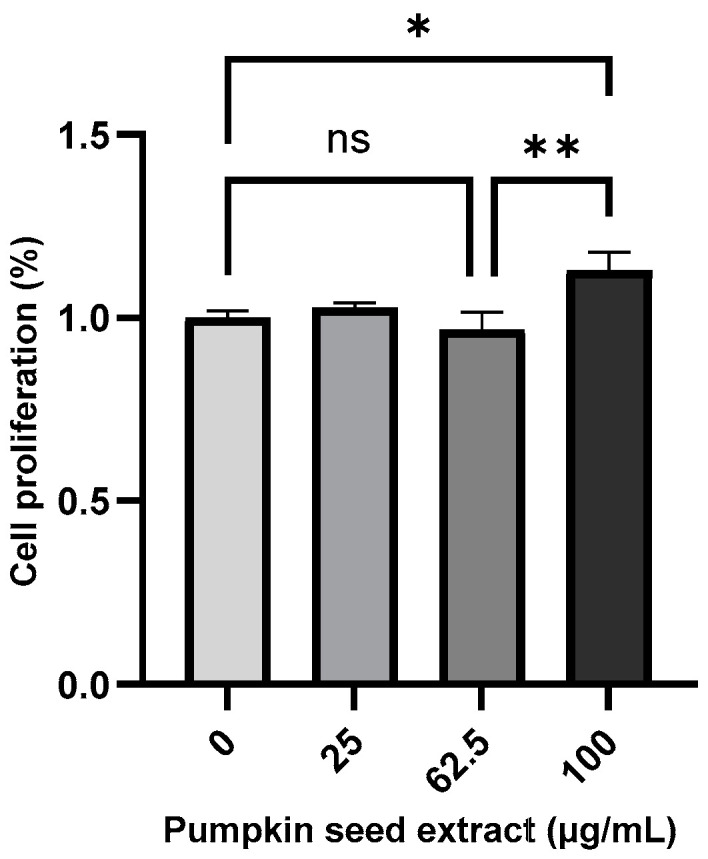
Proliferation of Saos-2 cells treated with pumpkin seed extract (0, 25, 62.5, and 100 µg/mL). Data are expressed as the mean ± SD (** *p* < 0.01, * *p* < 0.05). ns: non-significant.

The effect of pumpkin seed extract on the proliferation of Saos-2 cells was measured using the MTT assay (Figure 4), which revealed a significant increase in the proliferation of cells treated with 100 µg/mL extract (*p* < 0.05).

### 3.5. Effect of Pumpkin Seed Extract on the ALP Activity

**Figure 5:**
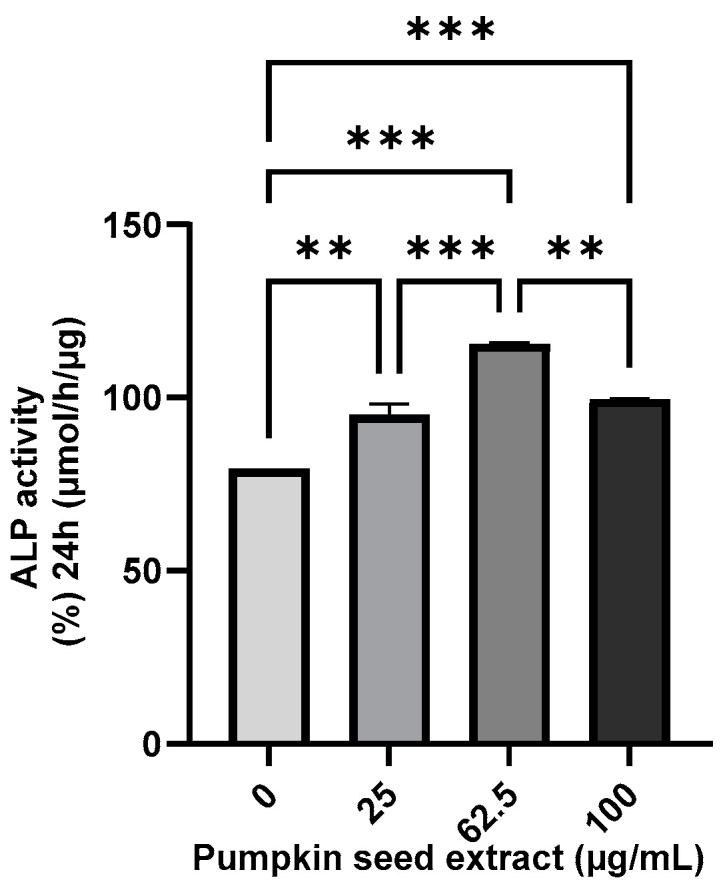
Alkaline phosphatase (ALP) activity in Saos-2 cells treated with pumpkin seed extract (0, 25, 62.5, and 100 µg/mL). Data are expressed as the mean ± SD (*** *p* < 0.001, ** *p* < 0.01).

The ALP activity of the pumpkin seed extract was analyzed using an ALP assay, which revealed that compared with the non-treated cells, there were significant increases in ALP activity in Saos-2 cells treated with all assessed extract concentrations, with the highest activity being detected in cells treated with the 62.5 µg/mL extract (*p* < 0.001) (Figure 5).

### 3.6. Effects of Pumpkin Seed Extract on Body Weight, Body Weight Gain, Food Intake, and Food Efficiency Ratio

**Figure 6:**
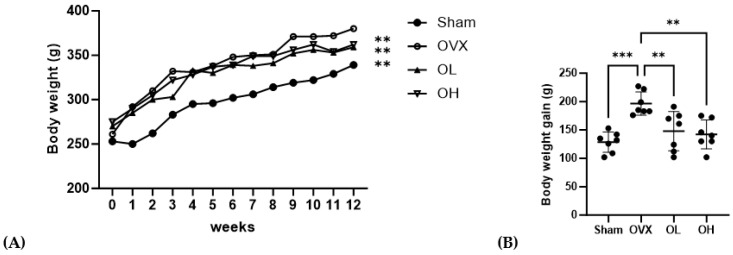
Effects of pumpkin seed extract on rat body weight and body weight gain. (**A**) Body weight. (**B**) Body weight gain. Data are expressed as the mean ± SD (*** *p* < 0.001, ** *p* < 0.01 vs. OVX). Sham, Sham-operated control group; OVX, ovariectomized negative control group; OL, OVX + low dose of pumpkin seed extract (250 mg/kg b.w.); and OH, OVX + high dose of pumpkin seed extract (500 mg/kg b.w.).

The b.w., b.w. gain, food intake, and FER data are shown in Figure 6 and Table 3. The initial b.w. of the rats was not significantly different among the groups. After 12 weeks of oral administration of pumpkin seed extract, OVX group showed significantly higher final b.w. (*p* < 0.01) and b.w. gain (*p* < 0.01) than the other groups. The groups administered pumpkin seed extract (OL and OH) showed no significant difference in the final body weight and b.w. gain (*p* = 0.000) compared with the Sham group. The food intake and FER did not differ compared to OVX throughout the 12-week of administering the pumpkin seed extract.

### 3.7. Effects of Pumpkin Seed Extract on ABF, UF, BAT, and SCF Weight

**Figure 7:**
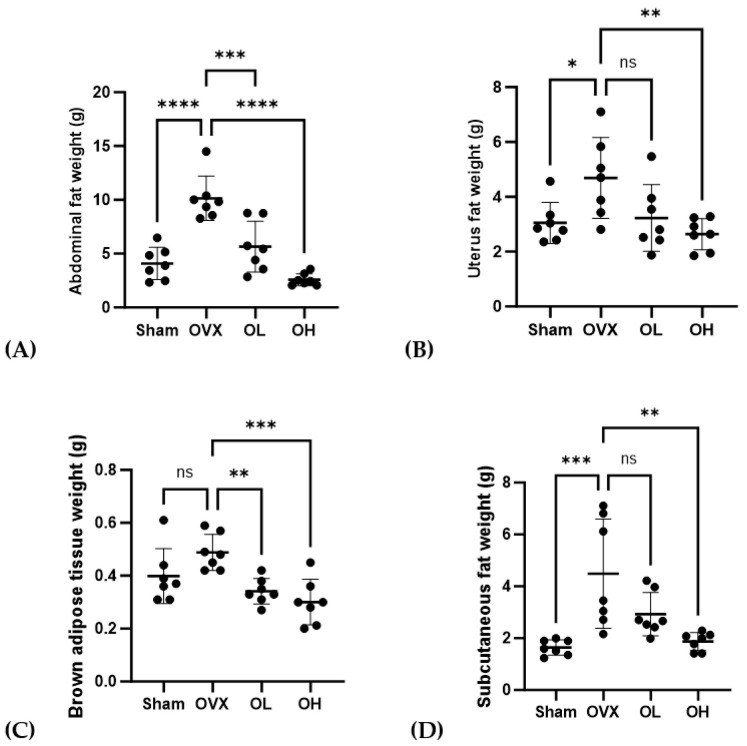
Effects of pumpkin seed extract on the weight of abdominal fat (ABF), uterine fat (UF), brown adipose tissue (BAT), and subcutaneous fat (SCF). (**A**) ABF weight. (**B**) UF weight. (**C**) BAT weight. (**D**) SCF weight. The data are expressed as mean ± SD (**** *p* < 0.0001, *** *p* < 0.001, ** *p* < 0.01, * *p* < 0.05 vs. OVX). ns: non-significant.

The weights of ABF, UF, BAT, and SCF are shown in Figure 7. OVX group had the highest ABF weight (*p* < 0.001). OVX group had significantly higher weights of UF and SCF (*p* < 0.05, *p* < 0.01) than those of Sham and OH groups and significantly higher weights of BAT than those of OL and OH groups (*p* < 0.01). The OH group showed a significantly lower fat weight than the OVX group (*p* < 0.01).

### 3.8. Effects of Pumpkin Seed Extract on Serum Lipid Profile

**Figure 8:**
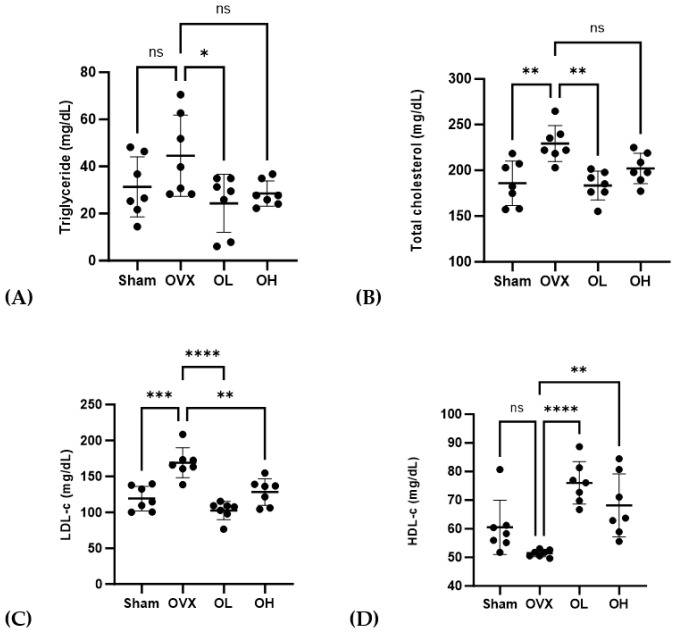
Effects of pumpkin seed extract on serum lipid profiles. (**A**) Triglycerides. (**B**) Total cholesterol. (**C**) Low-density lipoprotein-cholesterol (LDL-c). (**D**) High-density lipoprotein-cholesterol (HDL-c). Data are expressed as mean ± SD (**** *p* < 0.0001, *** *p* < 0.001, ** *p* < 0.01, * *p* < 0.05 vs. OVX). ns: non-significant.

The serum lipid profiles of treated rats are shown in Figure 8. Rats in the OVX group were found to be characterized by significantly higher levels of TG (*p* < 0.05), TC (*p* < 0.01), and LDL-C (*p* < 0.01), whereas compared with these rats, those in the OL group had significantly lower levels of TG, TC, and LDL-c. Among the ovariectomized groups, serum HDL-C levels were the lowest (*p* < 0.01) in the OVX rats, whereas those in the group treated with the pumpkin seed extract were characterized by a significant increase in serum HDL-C levels (*p* < 0.01).

### 3.9. Effects of Pumpkin Seed Extract on Atherogenic Index (AI) and Cardiac Risk Indices (CRI-I and CRI-II)

**Figure 9:**
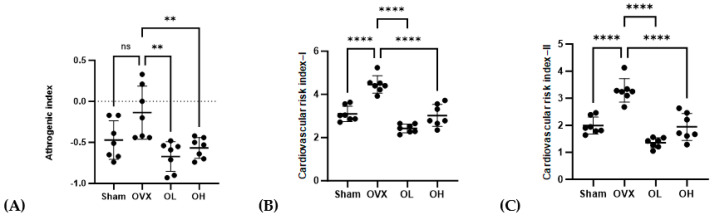
Effects of pumpkin seed extract on atherogenic index (AI) and cardiovascular risk indices (CRI−I and CRI−II). (**A**) AI. (**B**) CRI−I. (**C**) CRI−II. Data are expressed as the mean ± SD (**** *p* < 0.0001, ** *p* < 0.01 vs. OVX). ns: non-significant.

The AI, CRI-I, and CRI-II values are shown in Figure 9. The OVX group showed the highest values for AI, CRI-I, and CRI-II (*p* < 0.01) among all groups. The OL group showed the lowest values for CRI-I and CRI-II among all groups. There were no significant differences in AI, CRI-I, or CRI-II between the OL and OH groups.

### 3.10. Effects of Pumpkin Seed Extract on Hepatic Function Levels

**Figure 10:**
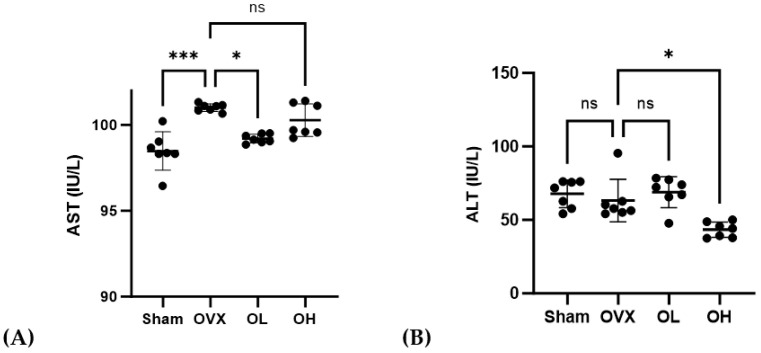
Effects of pumpkin seed extract on hepatic function. (**A**) AST. (**B**) ALT. The data are expressed as mean ± SD (*** *p* < 0.001, * *p* < 0.05 vs. OVX). ns: non-significant.

The AST and ALT levels in the serum are shown in Figure 10. The OVX group showed higher AST levels than those of the Sham and OL groups (*p* < 0.001, *p* < 0.05). The OH group showed the lowest ALT levels among all groups (*p* < 0.05).

### 3.11. Effects of Pumpkin Seed Extract on an Oxidative Stress Marker in Serum

**Figure 11:**
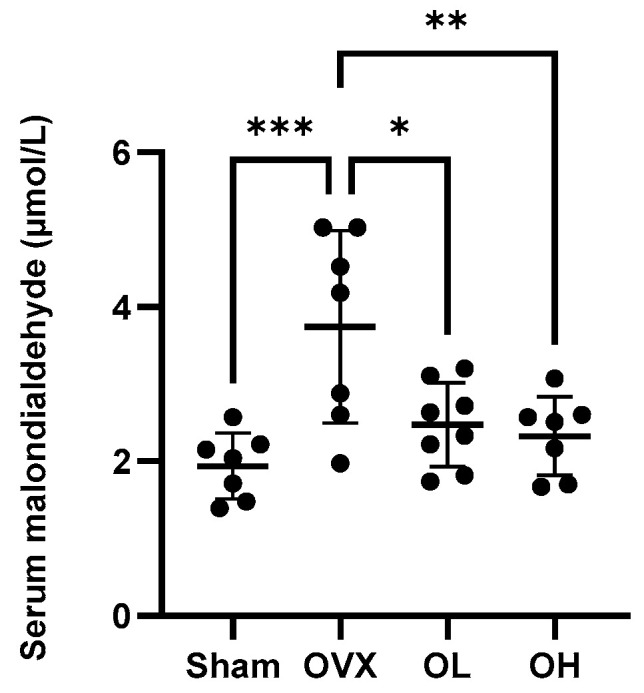
Effects of pumpkin seed extract on the serum levels of malondialdehyde. The data are expressed as the means ± SD (*** *p* < 0.001, ** *p* < 0.01, * *p* < 0.05 vs. OVX).

Values obtained for the serum levels of the oxidative stress marker MDA are shown in Figure 11. Among the assessed groups, the highest levels of MDA were detected in the sera of OVX group rats (*p* < 0.05), whereas in contrast, there were significant reductions in serum MDA levels in rats in the pumpkin seed extract-treated OL and OH groups.

### 3.12. Effects of Pumpkin Seed Extract on Estrogenic Safety Function in the Uterus

**Figure 12:**
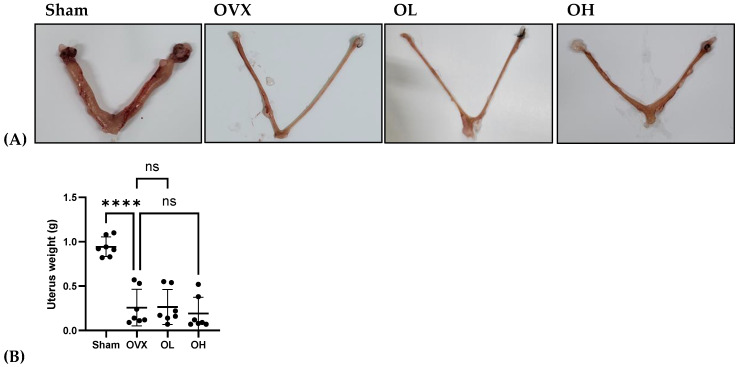
Effects of pumpkin seed extract on estrogenic safety in the uterus. (**A**) Representative images of the uterus. (**B**) Uterus weight (g). The data are expressed as the means ± SD (**** *p* < 0.0001 vs. OVX). ns: non-significant.

The results of the estrogenic safety function in the uterus are shown in Figure 12. The rats in all ovariectomized groups were characterized by very low uterus weights. This also indicates the success of the ovariectomy.

### 3.13. Antiosteoporotic Effect of Pumpkin Seed Extract in the Femur

**Figure 13:**
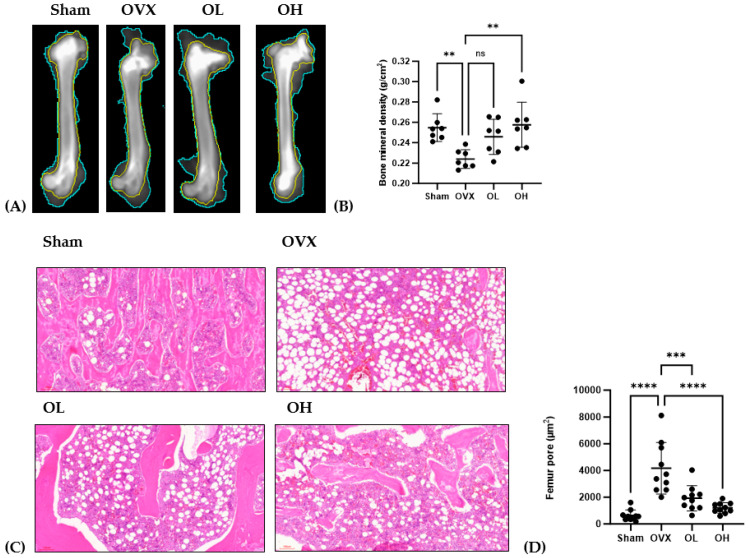
Antiosteoporotic effects of pumpkin seed extract in the femur. (**A**) Dual-energy X-ray image of the femur. (**B**) Bone mineral density. (**C**) Hematoxylin and eosin-stained femur sections. (**D**) Femur pore size. The data are expressed as means ± SD (**** *p* < 0.0001, *** *p* < 0.001, ** *p* < 0.01 vs. OVX). ns: non-significant.

The antiosteoporotic effects of pumpkin seed extract on the femur are shown in Figure 13. We detected a significant reduction in BMD among rats in the OVX group (*p* < 0.01), whereas in contrast, those in the OH group were characterized by significant increases (*p* < 0.01). Among the assessed groups, the femurs of rats in the OVX group were characterized by significantly larger pore sizes than those in the femurs of rats in the other groups (*p* < 0.001), with the femurs of rats in the OH group having the smallest pores.

### 3.14. Effects of Pumpkin Seed Extract on the mRNA Levels of Antiaging Genes in the Liver

**Figure 14:**
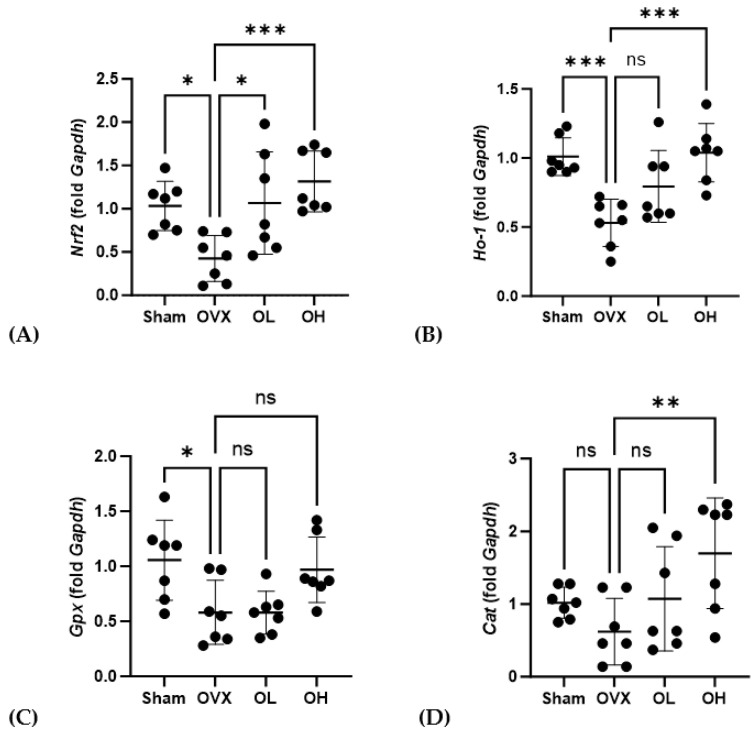
Effects of pumpkin seed extract on the mRNA expression of antiaging genes in the liver. (**A**) Nuclear factor erythroid 2-related factor (*Nrf2*). (**B**) Heme oxygenase-1 (*Ho-1*). (**C**) Glutathione peroxidase (*Gpx*). (**D**) Catalase (*Cat*). The glyceraldehyde-3-phosphate dehydrogenase (*Gapdh*) gene was used as an internal control. Data are expressed as the mean ± SD (*** *p* < 0.001, ** *p* < 0.01, * *p* < 0.05 vs. OVX). ns: non-significant.

Data obtained for the mRNA expression of antiaging-related genes in the liver are presented in Figure 14. After 12 weeks of oral administration of pumpkin seed extract, OL and OH group rats were found to have higher levels of *Nrf2* than those of OVX group rats (*p* < 0.05 and *p* < 0.001, respectively), and compared with the OVX group, we detected significantly higher levels of *Ho-1* and *Cat* expression in the OH group (*p* < 0.001 and *p* < 0.01).

### 3.15. Effects of Pumpkin Seed Extract on the mRNA Expression of Cardiovascular Protective Genes in the Liver

**Figure 15:**
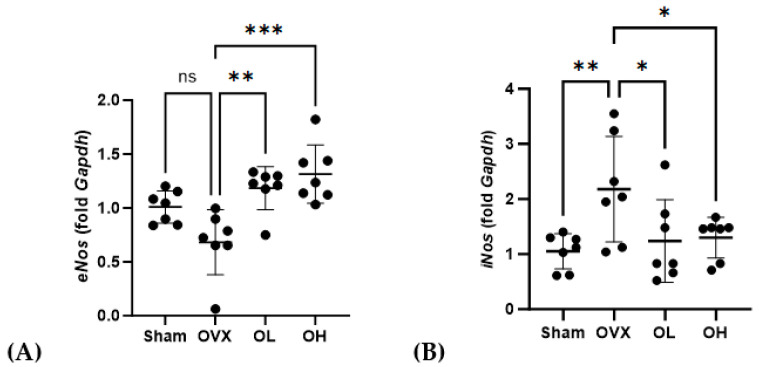
Effects of pumpkin seed extract on mRNA expression of cardiovascular protective genes and NO levels in the liver. (**A**) Endothelial nitric oxide synthase (*eNos*). (**B**) Inducible nitric oxide synthase (*iNos*). The glyceraldehyde-3-phosphate dehydrogenase (*Gapdh*) gene was used as an internal control. Data are expressed as the mean ± SD (*** *p* < 0.001, ** *p* < 0.01, * *p* < 0.05 vs. OVX). ns: non-significant.

Data obtained for the mRNA expression of cardiovascular-protective genes in the liver are shown in Figure 15. Among the ovariectomized rats, *eNos* mRNA levels in the OL and OH groups were significantly higher than those in the OVX group (*p* < 0.01 and *p* < 0.001, respectively), whereas in contrast, the levels of *iNos* expression in the OL and OH groups were significantly lower than those in the OVX group (both *p* < 0.05).

### 3.16. Effects of Pumpkin Seed Extract on mRNA Expression of Brown Adipogenesis Genes in BAT

**Figure 16:**
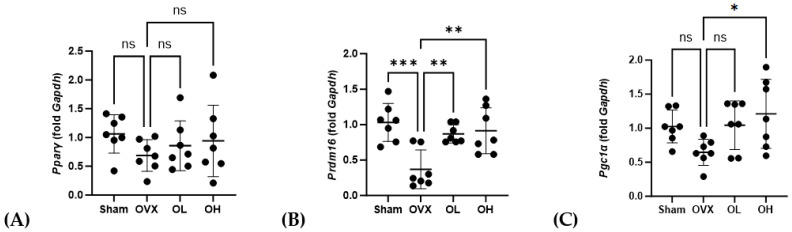
Effects of pumpkin seed extract on mRNA levels of genes related to brown adipogenesis in brown adipose tissue. (**A**) Peroxisome proliferator-activated receptor gamma (*Pparγ*). (**B**) PR/SET domain 16 (*Prdm16*). (**C**) Peroxisome proliferator-activated receptor-gamma coactivator (*Pgc1α*). The glyceraldehyde-3-phosphate dehydrogenase (*Gapdh*) gene was used as an internal control. Data are expressed as the mean ± SD (*** *p* < 0.001, ** *p* < 0.01, * *p* < 0.05 vs. OVX). ns: non-significant.

The mRNA levels of genes associated with adipogenesis in BAT are shown in Figure 16. With respect to *Pparγ*, we detected no significant differences in expression among the assessed groups. Contrastingly, compared with the OVX group, the levels of *Prdm16* expression were significantly higher in the OL and OH groups (*p* < 0.01). Similarly, the expression of *Pgc1α* in the OH group, although not the OL group, was significantly higher than that in the OVX group (*p* < 0.05).

### 3.17. Effects of Pumpkin Seed Extract on Thermogenesis in BAT

**Figure 17:**
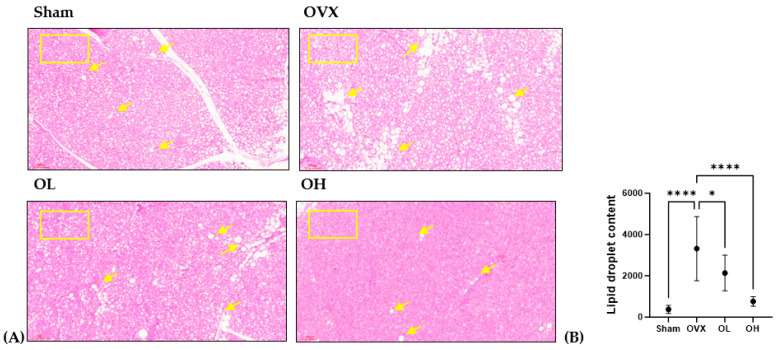
Effect of pumpkin seed extract on thermogenesis in brown adipose tissue (BAT). (**A**) Hematoxylin and eosin-stained BAT sections (×100). (**B**) Lipid droplets content in BAT. Arrows indicate lipid droplets. Data are expressed as the mean ± SD (**** *p* < 0.0001, * *p* < 0.05 vs. OVX).

The histological images of BAT are shown in Figure 17. BAT sections from the OVX group showed numerous lipid droplets and the largest droplets among all groups. The size of the fat droplets decreased in the BAT sections of the OL (*p* < 0.05) and OH (*p* < 0.0001) groups. As the square boxes indicate, the OH group showed a much browner area of brown adipose tissue than the OVX group.

### 3.18. Effects of Pumpkin Seed Extract on the mRNA Levels of Thermogenesis Genes in SCF

**Figure 18:**
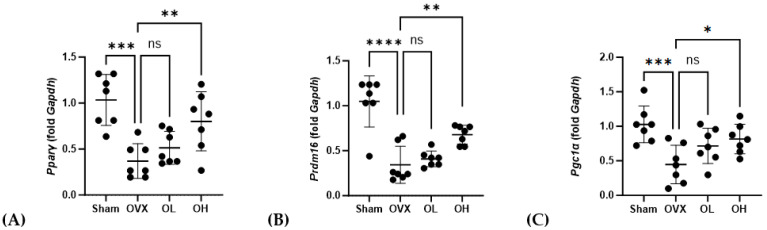
Effects of pumpkin seed extract on the mRNA levels of genes related to thermogenesis in SCF. (**A**) *Pparγ*. (**B**) *Prdm16*. (**C**) *Pgc1α*. The glyceraldehyde-3-phosphate dehydrogenase (*Gapdh*) gene was used as an internal control. Data are expressed as the mean ± SD (**** *p* < 0.0001, *** *p* < 0.001, ** *p* < 0.01, * *p* < 0.05 vs. OVX). ns: non-significant.

Data obtained for the levels of the mRNA expression of genes associated with thermogenesis in SCF are shown in Figure 18. Compared with rats in the OVX group, we detected no significant difference in the levels of *Pparγ* expression in the OL group, whereas these levels were significantly higher among the OH group rats (*p* < 0.01). For both *Prdm16* and *Pgc1α*, the levels of expression in the OH group were significantly higher than those in the OVX group (*p* < 0.01 and *p* < 0.05, respectively), although not in the OL group.

## 4. Discussion

Aging is the natural degeneration of physiological functions and the disruption of endocrine functions [1,2]. Menopause is an important stage in the life of females and is associated with aging, which results in a major deficiency of sex hormones, such as estradiol. Estradiol deficiency is associated with many diseases such as dyslipidemia, cardiovascular disease, hypertension, osteoporosis, vascular inflammation, and sleep disorders [4,5]. Few studies have focused on aging and metabolism, and most have dealt with either aging or menopause. However, all of these may be related. Clinical epidemiological studies have shown that estradiol deficiency increases the risk of cardiovascular diseases in which oxidative stress plays a crucial role [7]. A proper balance between oxidants and antioxidants is critical for the viability and proliferation of cells and organ functions [33]. Dietary antioxidants can slow the aging process and prevent related diseases by scavenging ROS and decreasing oxidative stress [34]. Because pumpkin seeds have been reported to be rich in phenolics and flavonoids and possess antioxidant properties [35], we designed the present study to demonstrate the effects of pumpkin seed extract in ameliorating osteoporosis and regulating lipid metabolism, cardiovascular diseases, and inflammation in Saos-2 cells and ovariectomized rats.

Phenolic and flavonoid compounds are secondary metabolites produced by plants that perform various important functions [36]. They provide defense against various diseases in plants [37]. In the present study, we obtained values of 784.61 ± 7.94 mg GAE/g and 0.70 ± 0.18 mg QE/g for total polyphenol and total flavonoid contents, respectively, in a 100 mg/mL preparation of pumpkin seed extract. Antioxidant activity involves the use of compounds or mixtures to reduce pro-oxidants, free radicals, or reactive species [38]. Relative to ascorbic acid, we obtained values of 95.31% ± 0.42% and 92.67% ± 0.20% for DPPH and ABTS radical-scavenging activities of the pumpkin seed extract, respectively. These results clearly show that both antioxidants, total polyphenol and flavonoid, and antioxidant activities of DPPH and ABTS in pumpkin seed extracts may have better antiradical and antioxidant activities. In addition to the phenolic and flavonoid compounds, pumpkin seeds are a rich source of unsaturated fatty acids and essential fatty acids, such as oleic acid (Omega-9), linoleic acid (Omega-6), and alpha-linolenic acid (Omega-3) that contribute to hormone synthesis and immune system regulation. Moreover, essential fatty acids have been established to play beneficial roles associated with reductions in cardiovascular morbidity and mortality and the prevention of cancer, arthritis, and hypertension, which are highly associated with the menopausal period [39].

To validate the positive effects of pumpkin seed extract, we performed in vitro and vivo studies using Saos-2 cells and OVX group treated with two different concentrations of the seed extract.

An increase in body and fat weight can be associated with a disordered energy metabolism attributable to a deficiency of estrogens [40]. By promoting adipocyte differentiation, estrogens have been established to have a pronounced effect on the regulation of the lipid metabolism, insulin resistance, and inflammatory activity in the body, and in this regard, we found that 12 weeks of treatment with pumpkin seed extract had the effect of lowering the body weight and body weight gain of ovariectomized rats [41]. To evaluate the toxicity of the treatment, we analyzed the serum levels of AST and ALT, which are important serum biomarkers of hepatic function, and increases in these levels are considered to be indicative of liver damage [42]. Although AST is mainly present in the cytoplasm of hepatocytes, AST is found in the cytoplasm and mitochondria of the heart, liver, and kidneys. We accordingly assessed the effects of pumpkin seed extract in the different rat groups by determining the levels of these hepatic markers of liver function, with the results indicating that menopause can influence the levels of AST and ALT, and treatment with pumpkin seed extract can rectify the abnormal levels of these enzymes; the normal range of AST and ALT has been reported in a previous study [43].

Both aging and the menopausal period are associated with increases in oxidative stress and excessive ROS production, which are deleterious to liver and cardiovascular functions [42]. Pumpkin seed extract ameliorates oxidative stress caused by menopause and aging, playing an antioxidative role in cardiovascular diseases, triglyceride accumulation in the body, and dysfunction in energy generation [42,44]. Consistently, in the present study, we found that the administration of pumpkin seed extract contributed to a reduction in the levels of lipid peroxidation, as assessed by an analysis of serum MDA levels [45]. *Nrf2* is a transcription factor that regulates cellular redox status and is involved in the maintenance of homeostasis; *Nrf2* levels are upregulated under oxidative stress but are reduced with aging [46]. *Ho-1* protects cells from oxidative stress, and its levels are reduced due to estrogen deficiency during menopause [47,48]. *Cat* is an antioxidant enzyme that reduces oxidative stress by catabolizing ROS [49]. In the present study, we evaluated the mRNA expression of these enzymes in ovariectomized rats treated with pumpkin seed extract, and accordingly detected the upregulated expression of all three enzymes, which would thereby contribute to the protection of cells from oxidative stress and the enhancement of the catabolism of ROS.

Both aging and menopause increase the risk of cardiovascular diseases [50,51]. Oxidative stress during aging leads to inflammation in the endothelium, resulting in cardiovascular diseases, such as hypertension, stroke, and atherosclerosis [52]. Dyslipidemia, an imbalance in the levels of TG, TC, LDL-c, and HDL-c, is characteristic of aging, and is a cardiovascular risk factor [53,54]. Estrogen plays a protective role against aging-related diseases by regulating lipid metabolism [55], and estrogen deficiency increases postmenopausal dyslipidemia [56]. In the present study, the OVX group showed the highest levels of TG, TC, and LDL-c and the lowest level of HDL-c among all groups, which is in agreement with the lipid profile of menopausal women [57]. OVX-induced changes in the lipid profile were significantly reduced in the OL and OH groups, indicating the beneficial effects of pumpkin seed extract. Moreover, in response to a reduction in the levels of LDL-c and increase in the levels of HDL-c, we detected more favorable values of AI, CRI-I, and CRI-II, which serve as indices of cardiovascular risk. In the vascular system, estradiol mediates *eNos* activation by binding to estrogen receptors, resulting in NO generation. NO plays a key role in the relaxation of blood vessels; therefore, *eNos* activity is involved in vasodilation [58,59]. Owing to the decreased estrogen levels during menopause and aging, the levels of *eNos* and NO decrease, increasing the risk of cardiovascular diseases. The expression of *iNos* under inflammatory conditions results in the production of large amounts of NO, which can result in atherosclerosis [60,61], and in the present study, we demonstrated that pumpkin seed extract can contribute to protecting the vascular system by upregulating *eNos* in vasodilation and downregulating *iNos* under inflammatory conditions. Based on these results, we believe that pumpkin seed extract protects against cardiovascular diseases.

Osteoporosis is associated with an imbalance in the relative numbers of osteoclasts and osteoblasts, in which there are larger numbers of osteoclasts removing older bone cells than there are osteoblasts generating new cells [62]. Given that reductions in the levels of calcium and hormones can lead to reductions in bone mineral content and density and general poor bone health, these parameters would also be adversely affected by the elevated levels of oxidative stress [63,64]. In this context, we found that treatment with pumpkin seed extract promoted an increase in the activity of ALP, which serves as an indicator of osteogenesis, thereby providing evidence to indicate that the antioxidants present in this extract may have an antiosteoporotic effect by enhancing osteoblast differentiation. Consistent with our observation of an increase in osteoblast function of Saos-2 cells treated with pumpkin seed extract, we detected significant increases in the bone mineral density of rats in the OL and OH groups compared with those in the OVX group. Moreover, compared with rats in the OVX group, we detected significant reductions in the sizes of pores in the femurs of OL and OH group rats.

By causing a reduction in brown adipose thermogenesis, menopause and aging are typically associated with a dysregulation of core body temperature, thereby resulting in obesity and a disruption of homeostasis [65]. Thermogenesis is an essential process for maintaining body temperature and function. Heat production mostly occurs in BAT; however, the browning of WAT is also involved in thermogenesis [66]. Unlike WAT, which stores energy as triglycerides, BAT and beige or brown adipocytes utilize fatty acids and glucose to synthesize ATP and produce energy in the form of heat through nonshivering mechanisms [67]. *Pparγ* downregulates the inhibition of browning- and thermogenesis-related gene expression [68]. *Prdm16* plays an important role in the browning and thermogenesis of adipocytes and promotes the induction of *Pgc1α* transcription, which affect lipid and bone metabolism [69,70]. We found that by inducing the upregulated expression of *Prdm16* and promoting the induction of *Pgc1α* in both BAT and SCF, treatment with pumpkin seed extract contributed to an increase in brown adipose thermogenesis. The decrease in circulating estrogen levels during menopause increases the fat mass, metabolic dysfunction, and temperature regulation [71]. This may affect thermogenesis by downregulating the expression of *Pparγ*, *Prdm16*, and *Pgc1α*, thereby reducing energy and heat production, which in turn contribute to obesity and diminished metabolism associated with menopause [72].

Previous studies have shown that pumpkin seed extract may exert antioxidant effects by reducing oxidative stress [73] and anti-obesity effects by lowering lipid content and insulin resistance in obese rats [74] and may ameliorate menopausal symptoms related to blood pressure, uterine endometrial thickness, serum lipids, and bone density in ovariectomized rats [73,74]. In the present study, our findings indicate that pumpkin seed extract has antioxidant effects and can contribute to mitigating the adverse effects of menopause with respect to antioxidant activity, body weight, oxidative stress, serum lipids, cardiovascular diseases, osteoporosis, and thermogenesis.

## 5. Conclusions

Collectively, our findings in this study have revealed that pumpkin seed extract enhances ALP activity in Saos-2 cells and mitigates ovariectomy-induced weight gain, oxidative stress, dyslipidemia, cardiovascular disease, inflammation, and osteoporosis, mediated via its antioxidant properties. However, because menopause and aging are complicated processes that are linked to several inter-related diseases, more in-depth mechanistic studies must be conducted to fully comprehend the benefits of pumpkin seed extract. Although several studies have focused on aging and menopause in isolation, little research has been conducted to establish correlations between the two. Thus, further research on the metabolism of antioxidants in aging- and menopause-related diseases is warranted. This study provides substantial data for clinical studies to further establish the efficacy of pumpkin seed extract in the treatment of aging- and menopause-related diseases.

## Figures and Tables

**Table 1:** Total polyphenol and flavonoid contents in pumpkin seed extract.

Total Polyphenols (mg GAE/g)	Total Flavonoids (mg QE/g)
784.61 ± 7.94	0.70 ± 0.18

**Table 2:** Chemical compounds identified in pumpkin seed extract.

	Peak Name	Retention Time (min)	Compound Name	Relative Area%
**1**	C15	20.110	*cis*-10-Pentadecenoic acid	12.83
**2**	C16	21.866	Palmitic acid (hexadecanoic acid)	17.47
**3**	C18	26.449	Stearic acid (octadecanoic acid)	5.79
**4**	C18 1n9c	27.380	Oleic acid	23.40
**5**	C18 2n6c	29.043	Linoleic acid	39.94
**6**	C183n3	31.071	Alpha-linolenic acid	0.23
**7**	C20	32.638	Arachidic acid (icosanoic acid)	0.34

**Table 3:** Body weight, body weight gain, food intake, food efficiency ratio, and organ weight of rats treated with pumpkin seed extract.

Group	Sham	OVX	OL	OH
Initial body weight (g)	253.33 ± 14.01 ^ns^	261.14 ± 33.12	270.82 ± 24.95	275.36 ± 21.00
Final body weight (g)	339.17 ± 29.39 **	380.83 ± 28.49	359.09 ± 47.36 **	362.55 ± 39.97 **
Body weight gain (g)	128.43 ± 17.92 ***	196.43 ± 20.43	147.86 ± 34.69 **	142.14 ± 25.48 **
Food intake (g/day)	17.27 ± 0.52 ^ns^	18.83 ± 0.48	17.64 ± 1.27	18.81 ± 0.38
FER (%)	9.23 ± 0.39 ^ns^	10.99 ± 1.24	7.77 ± 0.22	11.64 ± 2.58

Data are expressed as the mean ± SD (*** *p* < 0.001, ** *p* < 0.01 vs. OVX). ns: non-significant.

## Data Availability

The data presented in this study are available on request from the corresponding author. The data are not publicly available due to privacy.

## Supplementary Materials

The following supporting information can be downloaded at: https://www.mdpi.com/article/10.3390/antiox13020241/s1, Table S1: Sequences of primers used for qRT-PCR analysis.
