# Supplementary material for: Evaluation of Antioxidant Effects of Pumpkin (Cucurbita pepo L.) Seed Extract on Aging- and Menopause-Related Diseases Using Saos-2 Cells and Ovariectomized Rats
Source: Antioxidants (Basel). 2024 Feb 16;13(2):241. doi: 10.3390/antiox13020241 (PMC10886273; doi:10.3390/antiox13020241)
Supplement: Supplementary file 1 [file antioxidants-13-00241-s001.zip › antioxidants-2844056-supplementary.pdf]

Table S1. Sequences of primers used for qRT-PCR analysis

Target gene	Accession number	Forward (5′-3′)	Reverse (5′-3′)
Nrf2	NM_031789	CCATTTACGGAGACCCAC	TGAGCGGCAACTTTATTC
Ho-1	NM_012580.2	TGCTCGCATGAACACTCTG	TCCTCTGTCAGCAGTGCCT
Gpx	NM_030826.4	CTCTCCGCGGTGGCACAGT	CCACCACCGGGTCGGACATAC
Cat	NM_012520.2	GCGAATGGAGAGGCAGTGTAC	GAGTGACGTTGTCTTCATTAGCACTG
eNOS	NM_008713.4	ATGGATGAGCCAACTCAAGG	CTGTCCTCAGGAGGTCTTGC
iNOS	NM_012611.3	CTTGCCCCTGGAAGTTTCTC	GCACCTGGGGTTTTCTCCAC
Pparγ	NM_011146.3	CACCAACTTCGGAATCAGCTC	CAACCATTGGGTCAGCTCTTG
Pgc1α	NM_176075.2	TTGCCCAGATCTTCCTGAAC	TGAGGACCGCTAGCAAGTTT
Prdm16	NM_022687.2	AGACCGAGGACGGTATCCTG	TGGTATGGAGGACTCTCGCA
Gapdh	NM_017008.4	CCGTGTTCCTACCCCCAATG	GTTGCTGTTGAAGTCGCAGG

Abbreviations: *Nrf2*, nuclear factor erythroid 2-related factor; *Ho-1*, Heme oxygenase-1; *Gpx*, Glutathione peroxidase; *Cat*, Catalase; *eNos*, endothelial nitric oxide synthase; *iNos*, inducible nitric oxide synthase; *Pparg*, peroxisome proliferator-activated receptor gamma; *Pgc1α*, Peroxisome proliferator-activated receptor-gamma coactivator; *Prdm16*, PR/SET Domain 16; *Gapdh*, Glyceraldehyde-3-phosphate dehydrogenase.
